# PReS-FINAL-2347: Does switching anti-TNFΑ biologic agents an effective option in childhood chronic uveitis: the evidence from a systematic review and meta-analysis approach

**DOI:** 10.1186/1546-0096-11-S2-P337

**Published:** 2013-12-05

**Authors:** G Simonini, K Druce, R Cimaz, GT Jones, GT Macfarlane

**Affiliations:** 1 Rheumatology Unit-Department of Pediatrics, A.Meyer Children's Hospital University of Florence, Firenze, Italy; 2 Musculoskeletal Research Programme-Epidemiology Group, Institute of Applied Health Sciences, University of Aberdeen, Aberdeen, UK; 3 Rheumatology Unit-Department of Paediatrics, A.Meyer Children's Hospital,University of Florence, Firenze, Italy; 4 Musculoskeletal Research Programme-Epidemiology Group, Institute of Applied Health Sciences, University of Aberdeen, Aberdeen, UK

## Introduction

A subset of patients, of unknown percentage, fail to respond to TNFα blockers or are unable to tolerate these therapies and may therefore benefit from switching to another drug.

## Objectives

to report the evidence regarding the effectiveness of switching to another anti-TNFα treatment in children with childhood autoimmune chronic uveitis (ACU), failure/refractory to the first course of anti-TNFα treatment.

## Methods

A systematic search between January 2000 and May 2013 was conducted using EMBASE, Ovid MEDLINE, Evidence Based Medicine Reviews-ACP Journal Club, Cochrane libraries, and EBM Reviews. Studies investigating the efficacy of anti-TNFα therapy, as the second biologic treatment for ACU, in children (≤16 yrs) refractory to a first course of a single anti-TNFα treatment, topical and/or systemic steroid therapy and at least one DMARD, were eligible for inclusion. The primary outcome measure was the improvement of intraocular inflammation, as defined by the SUN working group criteria, at 6 (± 2) month follow-up on treatment.

## Results

Among 1086 identified articles, 128 were scrutinized: 10 observational studies, 7 on Adalimumab (ADA), 4 on Infliximab (INF), were deemed eligible, including 40 children (ADA n = 34, INF n = 6). JIA was the most common disease: 39 out 40 cases (97.5%).Seven-teen children received Etanercept: 11 were switched to ADA, the remaining 6 to INF. All the 23 children previously received INF were switched to ADA. Altogether, 30 children (24 on ADA, 6 on INF) out of 40 responded to treatment, and the proportion of participants with a positive response ranged from 43% to 100% individual studies.The pooled analysis suggested that a second anti TNFα treatment with ADA and INF has a favorable effect in the improvement of intraocular inflammation: 0.75 (95% CI: 0.67-0.81) was the combined estimate of the proportion of subjects improving All 6 children who received INF after a previous failure to a course of ETA, responded. Eight out of eleven (72.7%) children with a previous failure ETA, and 16 out 23 (69.5%) with a previous failure to INF, responded to a second course of anti TNFα treatment with ADA.Eighteen chidlren on ADA and all 6 on INF have been able to tape and/or discontinue systemic steroid administration; discontinuation/tapering of concomitant DMARD therapy was possible for 7 out of 19 children receiving ADA, 3 out of 4 children receiving INF. Four eligible papers did not report extractable data regarding visual outcome. Nine of 11 children (73%) showed improvement or stable normal visual acuity post ADA treatment, and 5 out of 6 children (79.3%) after INF. Among 33 anti-TNFα exposed children, data regarding side effects were not available from 2 studies (n = 7), 6 (25.2%) experienced adverse events: 5 while on ADA (all 5 complaining pain discomfort and/or local reactions), 1 on INF, who experienced a transient broncospastic cough.

## Conclusion

Switching between anti-TNFa obtains an overall probability of improvement of intraocular inflammation in 75% children affected by refractory ACU. No switching has been reported to ETA, all children received ADA or INF after the first anti TNFα failure.

## Disclosure of interest

None declared.

